# Not a Good Fix: Constitutivism on Value Change and Disagreement

**DOI:** 10.1007/s10670-023-00742-y

**Published:** 2023-10-13

**Authors:** Michael Klenk, Ibo van de Poel

**Affiliations:** Department of Values, Technology and Innovation, Jaffalaan 5, Delft, 2628 BX The Netherlands

**Keywords:** Value disagreement, Value change, Constitutivism, metaethics

## Abstract

We examine whether Thomsonian constitutivism, a metaethical view that analyses value in terms of ‘goodness-fixing kinds,’ i.e. kinds that themselves set the standards for being a good instance of the respective kind, offers a satisfactory explanation of value change and disagreement. While value disagreement has long been considered an important explanandum, we introduce value change as a closely related but distinct phenomenon of metaethical interest. We argue that constitutivism fails to explain both phenomena because of its commitment to goodness-fixing kinds. Constitutivism explains away disagreement and at best explains the emergence of new values, not genuine change. Therefore, Thomsonian constitutivism is not a good fix for realist problems with explaining value disagreement, and value change.

## Introduction

The Junkers G.38 was an excellent civil aircraft in the 1930s. As the world’s largest wheel-based plane, it carried up to 34 passengers in luxurious comfort. Nevertheless, it is not a good aircraft today. The G.38’s deafening noise and limited capacity would not fly with modern passengers. If only a trite bit of aerospace history, the case of the G.38 points to two distinct phenomena of metaethical significance: value disagreement and value change.

Value disagreement is usually thought of as a conflict of evaluative *convictions* (cf. Tersman 2021). For instance, convictions about the value of the G.38 today conflict with those held in the 1930s. Value change, in contrast, refers to a change in the actual *values* that our evaluative convictions purport to capture (cf. Swierstra, 2013). Both phenomena are distinct but related. From the value change perspective, changes in evaluative convictions need not be considered against a background of fixed values (as in an evaluative disagreement) but instead may reflect genuine changes in underlying values.

While value disagreement has long been considered to be of great metaethical significance, especially as a test for cognitivist value realism, the view that value judgements are truth-apt and at least sometimes true (cf. Rowland, 2017), value change has, in contrast, only recently garnered attention as an interesting metaethical explanandum in its own right (e.g. Swierstra, 2013; Baker, 2019; van de Poel, 2021).^1^

In this paper, we examine whether constitutivism offers plausible explanations of value disagreement and value change.^2^ Constitutivism explains values in terms of the constitutive features of *objects or acts of specific kinds* (Thomson, 2008), *valuers* (Lewis, 1989), *agents* or *deliberators* (Kant 1786 [1948]; Velleman, 2000; Korsgaard, 2008), *social practices* (Raz, 2003), or *God* (Zagzebski, 2004). Though constitutivism is a “broad church,” it is unified by a commitment to what Thomson (2008) called *goodness-fixing kinds* (Smith, 2018, p. 373). A goodness-fixing kind is a kind for which “what being [that kind] *is* itself sets the standards that a kind has to meet if it is to be good qua [that kind]” (Thomson, 2008, p. 21).^3^ Constitutivism, especially as a *realist* theory of value, has already become a “hot contender” in metaethics in recent years (Haase & Mayr, 2019), and Thomson’s (2008) influential account, in particular, has received much attention (cf. Wallace, 2011; Smith, 2010; Arneson, 2010; Bukoski, 2016; Lindeman, 2019).

But so far, nobody has critically analysed constitutivism’s potential to explain crucial aspects of value change and value disagreement. This is a significant omission: Prominent constitutivists like Thomson (2008) explicitly position their theory as a viable cognitivist, realist explanation of value change and value disagreement.^4^ A successful general explanation of both phenomena could thus endear constitutivism to non-constitutivist realists.

Therefore, we examine a cognitivist constitutivist explanation of value change and value disagreement. We show that constitutivism falls short in explaining both phenomena. Though both phenomena are distinct, constitutivism’s failure to explain them comes down to the same reason: its commitment to invariant goodness-fixing kinds, or so we argue.

In Sect. 2, we introduce the two key explananda – value disagreement and value change – in more detail, and illustrate their relation. In Sect. 3, we discuss the relevant features of constitutivism, focusing especially on goodness-fixing kinds. To keep the discussion manageable, we restrict our analysis and critique to Thomson’s (2008) influential rendering of realist constitutivism. Thomson has been most explicit and elaborated on the notion of a goodness-fixing kind, which, arguably, is a central feature of every constitutivist theory (Smith, 2018). Moreover, Thomson herself explicitly points to the problem of (diachronic) value disagreement and value change as explananda at several points in her exposition of constitutivism (cf. Thomson, 2008, pp 23 − 4, 40 − 3). In Sect. 4, we turn to our critical appraisal of Thomsonian constitutivist explanations of value disagreement and value change. We argue that it cannot offer a satisfying explanation of genuine disagreement: it implies that seemingly disagreeing parties do not really disagree but are simply talking past each other. Moreover, we argue that its commitment to goodness-fixing kinds makes constitutivism unable to explain genuine, continuous value change. In the final section, we comment briefly on the prospects for generalising our argument to other and all constitutivist theories.

Our analysis thus advances our understanding of constitutivism’s capability to deal with the challenges of value disagreement and value change. Therefore, it should be of interest to the debate about constitutivism and, more generally, to the debate about value disagreement and the emerging literature on value change. Although our conclusions vis-à-vis constitutivism will ultimately be negative, the exposition should be of some interest in itself, given the metaethical significance of value disagreement and value change and the virtual absence of evaluations of constitutivism’s in regards to those explananda.

### Key Explananda

#### Genuine Value Disagreement

In line with common usage in metaethics, we use the term ‘value disagreement’ to refer to opposing evaluative convictions (Tersman 2021).^5^ Typically, the type of disagreement that is taken to be of metaethical significance occurs synchronically. For instance, some people in the 1930s may have felt, in contrast with popular opinion, that the G.38 is not a good civil aircraft. But there is reason to also consider hypothetical disagreement and disagreement across time as significant for similar reasons (cf. Bogardus, 2016; Klenk, 2018) An example of the latter is the disagreement between people today and most people in the 1930s regarding the value of the G.38. In this broad, general sense, value disagreement is a phenomenon related to occurrent, past, or hypothetical mental states: it arises and wanes because people’s evaluative convictions change.

Our focus will be on the well-known metaethical challenge to explain how value disagreements can be *genuine*, at least in the sense that the people whose convictions make up the disagreement are not simply talking past each other (Hare, 1952; Tersman, 2006; Horn, 2020; Klenk, 2018, 2021).^6^

Consider Hare’s famous case of a tribe of cannibals that use the term ‘good’ as a general term of praise. To the consternation of the missionary visiting the tribe, they apply ‘good’ to people and actions that the missionary considers paradigmatically ‘bad.’ Brutal and rash people are ‘good’ for the cannibals, and compassionate, caring people ‘bad.’ Despite their differences in how they apply the term ‘good,’ their disagreement is genuine and should not be explained away as being merely verbal, or so the challenge goes (cf. Hare, 1952).

The challenge to explain the genuineness of value disagreement is a problem particularly for value realism, the view that value judgements are truth-apt and at least sometimes true (e.g. Enoch, 2011). There is a tension between value realism’s commitment to interpreting evaluative convictions as beliefs about the applicability of evaluative terms like ‘good’ and the pressure to interpret differences in the application of a term as evidence for differences in meaning (cf. Tersman, 2006). To begin with, note that value realism is *de facto* (though not logically) committed to value cognitivism,^7^ the view that value judgements are truth-apt. This means that value disagreement concerns opposing beliefs about the applicability of a term (e.g. ‘good’ does or does not apply to the G.38). But when two parties follow different rules for applying a term, then a probable interpretation is that they mean different things by that term.

The problem can be illustrated by focusing on a Davidsonian principle of charity (cf. Davidson, 2001).^8^ Following a charity principle, it would appear that people involved in a value dispute are talking past each other: they ascribe properties (cognitivism), but they follow different rules for applying a term, so they ascribe different properties (charity). The problem with value realism is that *genuine* value disagreement, conflicting convictions about the same object of reference, may vanish. Therefore, value realism is not a good explanation of value disagreement, or so the challenge goes (cf. Tersman, 2006, Klenk 2022).

There are several routes to salvage value realism. The existence of value disagreement as a descriptive, empirical fact could be challenged (cf. Klenk, 2019), the commitment to cognitivism could be abandoned (cf. Kahane, 2013), or alternative positions in the philosophy of language could be defended. However, some involve abandoning core commitments of value realism (cf. Tersman, 2006). In light of this challenge, it is promising to explore a constitutivist alternative. Before we do that, however, we introduce our second explanandum: value change.

### Continuous Value Change

At first sight, value change may seem closely related if not identical to value disagreement. For instance, our take on the G.38 today and that of people from the 1930s may simply be an evaluative disagreement over time. Indeed, value change has in the past often been described as changes in the mental states of (groups of) individuals. For example, there is sometimes a patterned change in evaluative convictions on a population level. Rescher (1967) called this “value redistribution,” that is, a “change in the extent or the pattern of distribution [of value convictions]” in a group (Rescher, 1967, p. 14).

So, one aspect of explaining value change is to explain how value redistribution patterns – of which the G.38 case is but an instance – come about. In other words, what accounts for changes in people’s evaluative convictions? Philosophers have only recently begun to devote attention to this issue, in debates about the causes of moral progress, moral revolutions, and emerging literature about the impact of new technologies on moral convictions (cf. Baker, 2019; Kitcher, 2021; Appiah, 2010; Klenk et al., 2022).

But value change is, at least conceptually, broader than value disagreement. It refers to changes in values themselves, rather than just changes in convictions about values.^9^ That sets it apart, conceptually but perhaps also substantially, from value disagreement. For some time already, in a diverse set of literature, including science and technologies studies, technology assessment, and the ethics of technology, scholars have suggested that technology changes values. For example, Bozdag and van den Hoven (2015) argue that social media and the impact of ‘filter bubbles’ on democracy have added weight to the value of transparency and diversity (see also Swierstra, 2013; van de Poel, 2021). Bublitz (2020) argues that informational technology and unprecedented abilities to infer people’s mental states change the value of privacy. Hopster et al. (2022) discuss several further cases that illustrate how technology has changed values, such as the demise of duelling among aristocratic men in early-modern Europe driven, at least in part, by new weaponry or the change in sexual mores related to the availability of contraceptives. Implicit in these discussions is a question about whether and how values themselves – rather than convictions about values – change over time. In the philosophical literature, Thomson’s discussion stands out because she explicitly affirms the possibility of value change when she claims that there are “cross-temporal” changes in values (Thomson, 2008: 20, see also pp 23ff).

For example, the fact that the G.38 today is a bad civil aircraft, while it was an excellent civil aircraft in the 1930s, may represent such a “cross-temporal” change in the value of the G.38, rather than merely a change in evaluative convictions.^10^ At a time there was consensus, at least among experts, that the Junker G.38 was an excellent civil aircraft, while experts today agree that it is not so good an aircraft. But today’s experts would not say that the experts who judged it to be an excellent civil aircraft in the 1930s were mistaken. This suggests that we do not have a case of value disagreement, but rather that the value of the Junkers G.38 has changed and that this value change explains the change in value convictions between then and now.

Therefore, value change as we understand it cannot be reduced to value disagreement. It is a meta-ethical explanandum on its own. In general, a suitable explanation should explain what it takes for values to change in a way that is not reducible to a change in evaluative convictions, and point to mechanisms that drive this change.^11^ We are particularly interested in a specific aspect of such an explanation. As we illustrate in Sect. 4.2, the challenge is not so much to explain how a *new* value comes into existence, but how an existing value changes over time. We will see that this raises difficult questions about the continuity of changed values across time below.^12^ Shedding some light on value change understood as a change in values (rather than value convictions) is, next to our primary aim of studying constitutivism, a significant contribution of the present article.^13^

### Thomsonian Constitutivism

We now briefly introduce four features of Thomsonian constitutivism that are relevant for the assessment of whether it can explain value change and disagreement.

First, Thomson holds that things can only ever be *good for* something, never good *simpliciter*. Standards of goodness are ultimately determined by goodness-fixing kinds. A goodness-fixing kind is a kind K_gf_ for which “what being a K_[gf]_*is* itself sets the standards that a K has to meet if it is to be good qua K_[gf]_” (Thomson, 2008, p. 21).^14^ Not all kinds are goodness-fixing. For example, Thomson suggests that pebbles, in contrast to artefacts like toasters or the G.38, or species like humans, are not *goodness-fixing* kinds. Thomson is very clear that there is only one possibility for a thing to be good as a member of some kind K if K is goodness fixing. As Smith (2018) notes, this commitment to goodness-fixing kinds, of some sort, to be the single source of evaluative standards is a crucial feature of constitutivism of various types. Not only are there good artefacts, like civil aircraft, but also good agents (Korsgaard, 2008), morally good acts (Thomson, 2008), and good ideal counterparts (Smith, 2013).^15^

Second, goodness-fixing kinds generate goodness orderings of things by what might be called the ‘standards’ of the respective goodness-fixing kind. For example, what a civil aircraft is *for* determines its standards, which determine an objective ordering of all things *as* civil aircraft.^16^ Importantly, these standards are independent of objective or subjective human interests (Thomson, 2011, p. 474).^17^ Thus, we can say that, for example, something is good *for England* by ranking objects in light of England’s goodness standards and the G.38 as a good aeroplane *for the 1930s* by narrowing the set of things that we rank according to the K_gf_ ‘civil aeroplane.’ Still, goodness standards exclusively depend on goodness-fixing kinds.

Third, when people make evaluative judgements (express their evaluative convictions, in the ‘loose’ parlance of Sect. 2), they ascribe goodness properties to the thing being evaluated. Thomson distinguishes several types of goodness properties that might be ascribed, but, for our discussion, two are particularly important (Thomson, 2011, pp. 26ff):

- Type (1) properties: The property of being good qua K_gf_, where K_gf_ is a goodness-fixing kind. For example, a toaster may be bad *as a toaster* because it always burns toast. Here we judge a toaster by the standards of the kind ‘toaster’, which is a goodness-fixing kind.
- Type (2) properties: The property of being good qua K_gf_ for a K. Here we judge a member of the kind K by the standards of the goodness-fixing kind K_gf_ relative to the kind K. For example, instead of a toaster, one might want to use a pan for toasting, and a frying pan might be good as a toaster *for a pan* (i.e., relative to other pans). Here K is the kind ‘frying pan’ and the goodness-fixing K_gf_ (by which we judge pans) are toasters. K does not need to be a goodness-fixing kind (like frying pans). For example, we may judge pebbles (not a goodness-fixing kind) for how good there are as chairs (e.g., for resting during a hike).

Fourth, Thomson (2008) endorses the epistemological claim that knowing a goodness-fixing kind means knowing what makes it good (Thomson, 2008, p. 35 − 6). The illustrates this idea with the example of an umbrella. An umbrella is a goodness-fixing kind and “we learn what being good *qua* umbrella is in the course of learning what being an umbrella is” (Thomson, 2008, p. 35). In learning what an umbrella is, we inevitably come to know, suggests Thomson, its standards for goodness. Though Thomson admits that it may be difficult to enumerate necessary and sufficient conditions for being a good umbrella (or, by extension, any goodness-fixing kind), she swiftly sets aside a fundamental sceptical worry about reliable epistemic access to these properties (cf. Thomson, 2008, p. 36, fn 2). Evaluative truths cannot be hard to grasp lest we admit that it is hard to grasp goodness-fixing kinds. But we all know what agents are, and civil aircraft, and umbrellas, or at least constitutivists like Thomson suggest that we do.

In summary, Thomsonian constitutivism suggests that values depend on the interest-independent standards set by goodness-fixing kinds. When we know a kind, such as ‘umbrella,’ we also know what makes it good, even though we need not be able to articulate its standard in detail. When we, finally, evaluate things, we can be taken as either evaluating something by the standard set by a goodness-fixing kind *in general* (type 1 ascription) or by the standard set by a goodness-fixing kind but *taking into account the subset of things belonging to some other kind*.

### Constitutivist Explanations of Value Change and Disagreement

So far, we discussed value change and disagreement and introduced constutivism’s key components. While the discussion of value disagreement drew on familiar discussions, our discussion of value change highlighted – from a metaethical perspective – a rather underappreciated phenomenon. Still, our claims drew on material familiar from adjacent debates.

In this section, we cover new ground. We examine constitutivist explanations of value change and value disagreement and argue that they fail to be satisfying. Notably, despite the metaethical significance of value disagreement and value change, and the increased interest in constitutivism, such a critical discussion has not been offered yet in the literature. Though constitutivism may bear promise as a metaethical theory for other reasons (cf. Smith, 2018), we argue that there are significant limitations to a Thomsonian constitutivist explanation of value disagreement and value change.

### Constitutivism on Value Disagreement

We argue that constitutivism cannot explain how evaluative disagreement is genuine. In short, because constitutivism is committed to goodness-fixing kinds, it has to interpret value disagreements as superficial disagreements where people talk past each rather than genuinely disagreeing.

The two types of property ascriptions allowed by Thomsonian constitutivism (see Sect. 3) allow for several potential explanations of value disagreement:

1) Two speakers may disagree because they judge the same thing by the standards of different K_gf_. Take for example a Rietveld chair, which is both a chair as well as a work of art, both (presumably) goodness-fixing kinds. One speaker may judge it as bad *as a chair*, the other as good *as a piece of art*.

2) Two speakers may refer to the same K_gf_ but disagree about the standards set by the K_gf_. For example, they may disagree about whether ‘originality’ is among the standards for pieces of art, and consequently, they may disagree about the goodness of e.g. a Rietveld chair as a piece of art.

3) Two speakers may refer to the same K_gf_ and agree about the standards set by K_gf_ but disagree about whether the thing being evaluated meets the standard. They may disagree about the goodness of a Rietveld chair as a piece of art because they disagree on whether it is aesthetically pleasing or not.

4) Two speakers may disagree because one ascribes a type (1) property while the other ascribes a type (2) property. For example, one speaker may judge the Junkers G.38 to be a bad civil aircraft (ascribing a type (1) property), while the other may judge it to be a good civil aircraft for an aircraft from the 1930s (a type (2) property, with K being aircraft from the 1930s and K_gf_ being civil aircraft).

5) Two speakers may disagree because they use different reference classes in ascribing type 2 -properties. For example, one speaker from the 1930s may use for the reference class K the aircraft known by the 1930s, while another speaker from the 2020s may use as reference class K aircraft known by the 2020s. Consequently, the first speaker may judge the Junkers G.38 as good and the second as bad. Their disagreement is explained by the ascription of different type (2) properties.

We will now discuss the five possible explanations of value disagreement that constitutivism has to offer. We suggest that only the first, fourth, and fifth can count as reasonable explanations of value disagreement from a constitutivist perspective.

The problem with the second and third explanations of disagreement is that the relevant kind of disagreement seems impossible, given that we consider only disagreement between relevantly informed parties as significant (see Sect. 2.1) and the constitutivist assumption that knowing a kind means knowing its standards (see Sect. 3). Consider the second explanation: disagreement is considered to be about the standards set by a goodness-fixing kind. Such disagreement seems impossible from a constitutivist perspective. To see why, consider the claims ‘The G.38 is a good aircraft’ and ‘The G.38 is not a good aircraft’ as seemingly incompatible claims about the standards set by the goodness-fixing kind ‘civil aircraft.’ Accordingly, one speaker may suggest that the standard for civil aircraft includes ‘low fuel consumption’ whereas the other speaker denies this. However, Thomson’s epistemic commitment to the transparency of goodness standards implies that, if both parties know the relevant kind, they could not be mistaken about its standards. So, assuming that we have two speakers that are informed in the sense that they know what a civil aircraft is, such a situation would not be possible, if constitutivism is true.^18^

According to the third explanation, disagreement concerns the question of whether an instance of a kind meets one of several possible standards fixed by the goodness-fixing kind. But, again, if we assume, in line with constitutivism’s epistemic claim and common assumptions about relevant types of disagreement, that both speakers are well-informed about the relevant descriptive facts of the G.38, such a disagreement cannot occur in the first place: both parties know about the relevant descriptive facts of the G.38 and thus they both grasp the goodness standard of the G.38 in virtue of knowing the goodness-fixing kind ‘civil aircraft.’^19^ Therefore, the relevant type of disagreement is, again, not possible from a constitutivist perspective.

In light of these observations, the relevant constitutivist explanation of value disagreement must depend on the first, fourth, or fifth potential explanation; disagreement about the goodness fixing kind in question, disagreement about whether a type 1 or type 2 property is ascribed, or disagreement about the reference class, or relevant kind, in ascribing type 2 properties. On any of these interpretations, however, genuine disagreement vanishes and the disagreement turns out to be merely verbal.

Suppose that we find that disagreement arises because speakers have a different K_gf_ in mind (in line with the first interpretation). Note that according to Thomsonian constitutivism, things are always good in a certain respect. Moreover, the theory does not privilege one respect over another. So, things may be good in one respect but bad in another. There is nothing mysterious about that for Thomsonian constitutivism. So in the case of the Rietveld chair, both speakers may be right: it is good *as a piece of art* and bad *as a chair, both of which are goodness-fixing kinds*. Alternatively, one speaker may judge the Rietveld chair good as a chair (a type 1 property) while another judges it as bad as a pebble (a type 2 property) (in line with the fourth interpretation). But that means that the disagreement is merely verbal. To see that, we could prompt the speakers to be more explicit and they would discover that, after all, they do not have a genuine disagreement about the Rietveld chair, but that they are simply talking past each other.^20^

Thompson (2008, pp. 40 − 3) herself seems to suggest suggests that seemingly disagreeing parties ascribe (different) type (2) properties when making judgements about goodness (in line with the fifth interpretation). For example, the disagreement about the G.38 will be interpreted as a disagreement about whether the G.38 is good in some respect, namely as an instance of a specific kind that cannot be the goodness-fixing kind ‘civil aircraft.’ But that explanation, too, explains away genuine evaluative disagreement. According to constitutivism, when people in the 1930s considered the G.38, they considered a particular kind, such as ‘civil aircraft from the 1930s’, and judged that within that kind, the G.38 was indeed a good civil aircraft, per the standards of the goodness-fixing kind ‘civil aircraft.’ When people today consider the G.38, they may consider another kind of object, such as ‘all civil aircraft, including those from the 1930s’ which includes modern planes like the Airbus A380, and they judge that the G.38 is not a good aircraft judged by the standard of a civil aircraft. Since speakers that know the kind in question cannot be interpreted as disagreeing about the very standard of the goodness-fixing kind (which, by assumption, they both know) their disagreement is naturally interpreted as merely verbal.

Therefore, given constitutivism, we would have to conclude that their disagreement is not genuine after all: seemingly disagreeing parties are merely talking past each other. For example, someone praising the G.38 as a good aircraft in the 1930s is simply not talking about the same thing as someone seemingly rejecting that claim when they ascribe a type 2 property that references a different kind that includes also modern aircraft. Therefore, constitutivism implies that evaluative disagreement is not genuine disagreement. Though people might have different attitudes in a given evaluative disagreement, e.g. concerning the G.38, the disagreement has vanished on a semantic level.^21^

In conclusion, constitutivism is at best on par with other realist theories of value with regard to the semantic disagreement challenge. Insofar as the genuine sense of disagreement felt in evaluative disagreements is a significant explanandum, constitutivism offers realists no advantage compared to rivaling non-cognitivist explanations.^22^

### Constitutivism on Value Change

As we noted above, value change is related but distinct from value disagreement and thus it presents a distinct explanatory challenge. In this section, we argue that constitutivism fails to satisfactorily explain value change. This is significant: Given that both challenges are independent, constitutivism may yet have gained ‘plausibility points’ (cf. Enoch, 2011) by successfully explaining value change. This may have partly offset the unsatisfactory explanation of value disagreement.

To illustrate the phenomenon of value change once again, it is instructive to briefly consider it in light of the different interpretations of value disagreement that we considered in the previous Sect. (4.1). Consider someone in the 1930s saying that the Junkers G.38 is a good plane. She may be interpreted as either ascribing a type-1 property, believing that planes will not much improve after the 1930s, or a type-2 property, with reference class planes from the 1930s (cf. Thomson, 2008, 42ff). An interpretation purely in terms of changing convictions would suggest that this value judgement may have changed by the 2020s either because another type 2-property is ascribed (i.e., with another reference class) or because by the 2020s it was clear that planes had – in fact - much improved and that the ascription of the type 1-property in the 1930s was mistaken. But it seems that the goodness of civil aircraft has also changed in another, substantive way since the 1930s: the value of the G.38 has changed. For example, today, one of the standards of good aircraft is sustainability, the environmental impact of civil aircraft should be as low as possible. Here, we seem to see a change in, in constitutivist terms, the standard set by a goodness-fixing kind. We shall now explore why Thomsonian constitutivism cannot explain such value change after all.^23^

The problem is that constitutivism can at best explain how *new* values come into being, but its commitment to goodness-fixing kinds prevents it from explaining genuine value change. In effect, it raises scepticism about the continuity of values.

The continuity of values refers to the fact that we perceive changed values as part of continuous development. What makes for a good civil aircraft may have changed between the 1930s and today (in constitutivist terms: the standards fixed by the kind have changed), but we still recognise them as standards for civil aircraft. For example, nowadays, it seems plausible to consider environmental emissions, and more generally sustainability targets, as one of the standards for good civil aircraft, while in the 1930s this was not yet among the standards for good civil aircraft. But they are still standards for civil aircraft (i.e., the goodness-fixing kind itself remains the same in relevant aspects). Therefore, we talk about changed rather than new standards. Similarly, what makes for a (morally) good leader or politician may have changed between the 1930s and today. However, we still recognise these changing standards as standards for leadership or political conduct. This means that an account of changing values must explain how the changed value is still recognisable as the value in question (see Jackman, 2020 for a related problem about concepts).

However, constitutivism cannot explain how values change. To explain how values change, constitutivism would have to explain how goodness-fixing kinds can change. But here constitutivism encounters two significant problems. Some terminology will be helpful to explore this point. Let the *goodness-set* of a goodness-fixing kind be a set of properties that account for the goodness of a particular instance of a goodness-fixing kind. For example, ‘being safe’ or ‘consuming little fuel’ may be a member of the goodness set for the goodness-fixing kind civil aircraft. Let the *goodness function* of a K_gf_ be a function that assigns properties to the goodness set of the goodness-fixing kind. Goodness sets are constituted by goodness functions. Therefore, our question becomes: Can constitutivism explain how (and why) goodness functions change?

#### Substantive Problems

In order to answer how constitutivism can explain how and why goodness functions change, it is natural to ask how a goodness function is determined in the first place. This would be to ask for what might be called a substantive explanation of (the constitution of) goodness functions.

However, substantive explanations of goodness functions are not available within constitutivist theories: goodness functions are assumed to be given and in that sense, they are primitive or ‘rock bottom’ for constitutivist theories. Naturally, different constitutivist theories will give very different answers as to what makes a goodness-fixing kind. But all have a hard time explaining what demarcates goodness-fixing kinds from non-goodness-fixing kinds. Some implicitly or explicitly maintain that goodness-fixing kinds are given as explanatory primitive (Smith, 2013; Korsgaard, 2008). Others employ further explananda like a Neo-Aristotelian idea of function or social practices to explain how goodness-fixing kinds are demarcated from non-goodness-fixing kinds (e.g. Thomson, 2008; Raz, 2003). But then the fact that functions or social practices ‘make’ a goodness-fixing kind is assumed as a theoretical, explanatory primitive. Thomson, for example, writes (Thomson, 2011, p. 473):

If an account could be produced that explained what made a kind be a kind such that what being a member is fixes what being a good member is—and thereby explained what makes the kind be a goodness-fixing kind—then that would be very welcome.

Constitutivists have not yet given an answer. On its own, that would perhaps not be damning, given that explanation has to stop somewhere. But since that particular explanation is required for a constitutivist account of value change, constitutivism falls short.

#### Formal Problems

There is also a formal reason to doubt that any, possibly forthcoming, substantive account of goodness functions can deliver an explanation of value change.

Value change often seems to occur because of relational factors. For example, subjective interests like an increased preference for sustainability or objective features like a decreasing supply of fossil fuels may suggest that sustainability becomes part of the goodness set of civil aircraft (cf. Morris 2015). But the goodness set is determined by a goodness function, which, according to constitutivism, is independent of such relational factors. After all, the kind *itself* sets its standards. What it means to function as a civil aircraft does not change because the world changes. Neither does a changed world change what it means to be a good agent and so on. Buying into that claim may be especially problematic when we consider artefacts (which is why we focused on the example of the G.38 throughout). After all, artefacts seem to have functions externally imposed by their designers (Oderberg, 2020, p. 122). Pace Oderberg, however, we can interpret the case of artefacts in light of constitutivist commitments. In that case, a goodness function may well *at some point* be influenced externally, but at that very point, the goodness-fixing kind and its goodness function are determined and fixed.

So, we can make sense of the idea that goodness-fixing kinds set their standards in the case of artefacts only by supposing that determination of a goodness function individuates the kind. That allows us to explain how design intentions (which depend on human interests) influence goodness functions in a way compatible with the constitutivist commitment that standards are independent of such interests. That explanation should carry over to non-artefact kinds, according to constitutivism. It means, however, that values cannot change. Of course, that does not rule out relational definitions of goodness functions per se, but it rules out all helpful definitions of that kind. Recursive relational functions along the lines of ‘Whatever properties are required to serve its function’ may be possible, but entirely unhelpful when we want to understand what goodness-fixing kinds are.

Therefore, if constitutivism maintains that goodness-fixing kinds set their ‘own’ standards, then goodness-fixing kinds cannot change. When values change, then we are dealing with new values (grounded in a different goodness function of a different goodness-fixing kind). But then we are not debating a value that has changed, but different things altogether.

In our view, this poses a significant challenge for realist constitutivists. But they may propose the following solution. They might argue that instances of a K_gf_ possess kind-relative goodness that is, somehow, the aggregation of the goodness with respect to what Thomson (2008) calls kind-relative virtues.

For example, consider the G.38 again and the K_gf_ civil aircraft. The K_gf_ gives us kind-relative virtues such as ‘long haul,’ ‘low energy consumption,’ and ‘great comfort.’ An *aggregation function* then determines how the kind relative virtues make up a good plane. We do not know this aggregation function but it may for example take the following form:

x is a good instance of K_gf_ iff for each of the relevant kind-relative virtues, x meets the minimum threshold for that virtue.

This interpretation allows for an alternative interpretation of type (2) properties above:^24^

x is a good K_gf_ for a K iff for each of the relevant kind-relative virtues fixed by K_gf_, x meets the relevant threshold set by K.

Crucially, while the goodness function of K_gf_ determines the goodness set, the K’s aggregation function determines whether or not an instance of K counts as a good K_gf_. This interpretation suggests a solution to the problem of value change. As we consider different kinds – such as planes from the 1930s – we are considering different thresholds for goodness. A plane from the 1930s like the G.38, perhaps, has a lower threshold for being a good aeroplane than a plane from the 2010s, like the A380.^25^

This interpretation adds nuance to the constitutivist picture. The ‘threshold’ interpretation thus suggests that the goodness of the G.38 is set not just by the goodness-fixing kind itself (civil aircraft) but by the (sub)kind K as well (,i.e. planes from the 1930s). This overcomes the objection we raised earlier as it allows us to say that people who praised at the time the G.38 as a good aircraft were indeed right, as the G.38 was, in this interpretation, a good aircraft for an aeroplane from the 1930s.

However, it raises another problem. It suggests that the goodness of the G.38 is actually fixed by two kinds, namely the goodness-fixing kind ‘civil aircraft and the kind ‘aircraft from the 1930s’. Constitutivism’s core distinction between goodness-fixing kinds and non-goodness-fixing kinds obstructs this interpretation. If sub-kinds (partly) determine goodness, then they are goodness-fixing and we have seen that such an account cannot satisfactorily explain value change above. If, however, we let go of the distinction between goodness-fixing kinds and non-goodness-fixing kinds then the interpretation we just considered may be possible, but it is no longer a (Thomsonian) constitutivist interpretation.

### Implications for Constitutivism in General

Whether our argument generalises from Thomson-style constitutivism to other forms of constitutivism depends on whether constitutivists can deviate from Thomson’s version of constitutivism on two points.

First, there is a question of whether constitutivism in general *must* explain at least some value disagreements as disagreements involving type (2) properties. We have provided a sound defence of this commitment conditional on Thomson’s epistemic claim that knowing a K_gf_ means knowing its goodness-fixing standards. By letting go of that Thomsonian claim, constitutivists, in general, may yet offer an analysis of disagreement that evades our objections vis-à-vis the semantic disagreement challenge.

Second, there is a question of whether constitutivism in general must hold on to the claim that goodness fixing kinds K_gf_ set their own standards for goodness independent of objective or subjective human interests. We stipulatively defined constitutivism, in line with Thomson, by a commitment to goodness fixing kinds that set their own standards for goodness. However, we have been silent on whether constitutivism, in general, could embrace such standards being modulated by *context*.

We cannot hope to resolve this problem here. But there is a simple idea. Namely that the attractiveness of constitutivism depends on the idea that it can draw on standards for goodness as somehow fixed, as Thomson says, independently of objective or subjective interests which purport to and promises to capture the intuition that moral truths, which also depend on truths about goodness, are “stance independent” (Shafer-Landau 2003).

Naturally, there is no logical barrier to weakening this commitment. For instance, latching onto recent work on functionalist accounts of morality (e.g. Klenk, 2021), constitutivists may hold that the standards are malleable in line with changing coordination problems. While such a view is possible, it would seem to bereft constitutivism of its distinct advantage over alternative realist views when it comes to accounting for the apparent objectivity of goodness and morality. Substantiating this claim, however, is a task for a further project.

## Conclusion

Thomsonian constitutivism’s status as a serious metaethical contender depends partly on its ability to explain relevant aspects of the metaethically significant phenomena of value disagreement and value change. We also argued that Thomsonian constitutivism fails to offer an explanation that is superior to realist alternatives. Therefore, value realists have to look elsewhere to successfully explain value disagreement and change. Apart from the detailed study of Thomsonian constitutivism vis-à-vis the challenges of value disagreement and value change, our paper more generally suggested the philosophical relevance of studying value change, in addition to value disagreement. In future work, more can be said about generalising the problem to other constitutivist accounts, the precise contours of value change, and its metaethical significance.

## Footnotes

1 Cognitivism as a semantic thesis about moral language is naturally thought of and combined with realism as a metaphysical thesis about moral facts, though there is no logical connection between both, cf. Kahane, 2013. Hence we explicitly present constitutivism as a cognitivist realist alternative.

2 As we discuss below in Sect. 2, we focus on specific *aspects* of these phenomena that need to be explained. Moreover, note that there is not *the* challenge from evaluative disagreement, or *the* challenge from value change. Different aspects of both phenomena raise different challenges. We specify the kinds of challenges we have in mind in Sect. 2.

3 Constitutivism’s unifying commitment to goodness-fixing kinds does not imply that normative standards governing individuals are explained by the nature of those individuals. Some constitutivists, like Korsgaard, think that there is but one goodness-fixing kind, agent, which fixes the standards for other kinds, while others, like Katsfanas 2013 and Velleman, are constitutivists in our sense only with regards to practical, not moral, standards.

4 Constitutivism may work for non-cognitivists, too; Ridge 2018. Nonetheless, constitutivism is naturally thought of as a realist alternative, which is what we will focus on in this paper.

5 We intentionally leave the term ‘conviction’ unspecified. How it is to be understood, precisely, will depend on questions about moral semantics and metaphysics that we will not address in this paper. It is generally understood that significant or relevant cases of evaluative disagreement do not depend on mistakes, such as factual mistakes or mistakes in reasoning, and occur amongst similarly well-informed speakers. So, we understand that the relevant type of disagreement concerns incompatible evaluative convictions held by similarly well-informed speakers that remain once such ‘mistakes’ have been clarified. Though this understanding of significant disagreement is common, and sufficient for our purposes, it is well-known that it is hard to specify, exactly, how to understand what amounts to a mistake or fault without precluding the possibility of interesting types of disagreement from the start (cf Kölbel, 2004).

6 Whether on has offered an explanation of value disagreement is thus, for the purposes of our paper, determined by whether one can explain the sense of genuine disagreement, per the semantic challenged outlined here. As we note elsewhere in the paper (e.g. fn 2 above), there is not *the* disagreement challenge and the explanation that we demand thus affects only a particular aspect of the phenomenon. Thanks to an anonymous referee to raise this question.

7 And neither is constitutivism; cf. Ridge 2018. Since our focus is on constitutivism as a cognitivist metaethical theory, the implication of non-cognitivism is a problem.

8 It should be noted that the Davidsonian principle of charity is not the only position in philosophy of language that can be used to argue that differences in application may indicate differences in meaning. It is possible to derive similar conclusions that make trouble for the value realist from several other prominent views in the philosophy of language, such as conceptual role semantics (see Wedgwood, 2001 for a discussion specifically in the context of moral terms) or various forms of social externalism (Putnam, 1975). This observation makes the challenge harder to overcome, as it does not rely on just one, possibly controversial, principle but instead can draw on a range of well-supported theories. Thanks to an anonymous referee for stressing this point.

9 Value change may thus enable a new novel explanation of value disagreement, at least when we consider diachronic value disagreement: the changed evaluative convictions reflect genuine changes in the values that our convictions purport to capture. This is one of the interesting ways in which both challenges may be connected.

10 As we show in Sect. 3, Thomson (2008) stresses that values are interest-independent and thus not reducible to what, roughly, we take to be valuable.

11 The literature on technologically-induced value change that we mentioned above has primarily focused on the latter, and proposed technology as a significant driver of value change. Less attention has been paid to the former question: in what ways do values change that is irreducible to a change in evaluative convictions?

12 Compared to the discussion of value disagreement, there is very little attention paid to the question how value themselves (rather than value convictions) can change. The focus has predominantly been on explaining how changing value convictions in some sense ‘track’ the values themselves (Street 2006, Enoch, 2010, Klenk, 2020). If value change is a real, however, such an explanation would be complicated by the fact that evaluative convictions would have to track dynamic, fluctuating values, which, arguably, complicates any such account.

13 It is worth emphasising that the explanatory challenges posed by value disagreement and value change are connected. Both challenges complement each by making demands about both ‘mind’ and ‘world’ aspects of a metaethical theory. The ‘mind’ aspects of metaethical theory require, amongst other things, a plausible semantic and epistemology that can explain the genuineness of value disagreement and how our evaluative convictions latch onto the values themselves. That ‘world’ aspect of a metaethical theory also requires, amongst other things, a theory of how, if at all, the things that make evaluative convictions correct can change. Notably, though both challenges are related, they are independent. A failure to explain value disagreement need not imply a failure to explain value change, and vice versa. Hence, both challenges offer the chance to score plausibility points (Enoch, 2011). Thus, if constitutivism could indeed offer a successful explanation of value change (and if value change is indeed metaethically significant, as we suggested above), it might have an advantage over rival realist theories after all.

14 What distinguishes goodness-fixing from non-goodness-fixing kinds? That is an important question. We briefly return to it in Sect. 4.2. Thomson, in any case, does not explicitly discuss how the set of goodness-relevant properties of a given K_gf_ are determined, though she mentions the kind’s function, design intentions (in the case of artefact kinds), and capacities as relevant possibilities, see Thomson, 2008, pp. 20–21.

15 Even if the justification for a constitutivist analysis of *moral* value does not simply follow from a constitutivist analysis of artefact value, cf. FitzPatrick, 2010, any constitutivist account must follow this schema.

16 As presented by Thomson, K_gf_ indeed allow for seemingly weird comparisons. For example, the K_gf_ ‘civil aircraft’ yields an objective ordering of e.g. Corinthian or Doric columns *as* civil aircraft. Whether this is an objection to the view, and whether it could be avoided, will not be of relevance for our argument and so we set these questions aside.

17 What distinguishes goodness-fixing from non-goodness-fixing kinds? As we discuss in Sect. 4.2, constitutivism assumes that there is a distinction, but does not itself explain how the distinction is determined. Thomson, for example, does not explicitly discuss how the set of goodness-relevant properties of a given Kgf are determined, though she mentions the kind’s function, design intentions (in the case of artefact kinds), and capacities as relevant possibilities (Thomson, 2008, pp. 20–21).

18 If, however, we have at least one uninformed speaker who does not know what a civil aircraft is, we do not have a case of relevant value disagreement. As discussed above, the disagreement would be not be relevant because it would depend on mistakes and occur amongst non-peers.

19 Again, perhaps one of the speakers is not well informed and that causes the disagreement. That is possible, of course, but then we do not have the relevant kind of disagreement since, as we discussed above, people surely disagree because some people are mistaken, or are not well-informed, but these cases of disagreement are generally discarded as irrelevant (cf. Klenk, 2018).

20 The same conclusion holds if, assuming speaker one ascribes a type 1 property, speaker two ascribes a type 2 property.

21 Of course, explaining apparent disagreements between aircraft enthusiasts may not strike one as a problem. But disagreements about morally good acts, or good politicians, or normative reasons will receive the same treatment and vanish in the constitutivist perspective. Of course, constitutivist could – like other realists – argue that conflicting attitudes persist and that they explain the sense of genuine disagreement about aircraft, politicians, or normative reasons. That, however, explanation, however, would seem to fit much better with a non-cognitivist approach to evaluative judgements in the first place (cf. Tersman, 2006).

22 Interestingly, while non-constitutivist realists may resolve the semantic disagreement challenge that we discuss by defending a suitable view in the philosophy of language that does not commit them to interpret differences in the application of a term as differences in meaning (see Sect. 2.1 above), constitutivist realism lacks this escape route. It succumbs to the disagreement challenge for reasons that are independent of semantic considerations. In that sense, constitutivism turns out to fare *worse* that non-constitutivist theories against the semantic disagreement challenge.

23 A seemingly obvious constitutivist explanation of value change can quickly be shown to fail. The suggestion may be that civil aircraft from the 1930 faired pretty poorly against the standards for the goodness-fixing kind ‘civil aircraft’, and the fact that they have tremendously improved technologically explains that modern civil aircraft fare much better against that same standard. That answer won’t do the explanatory work. When we account for value change in terms of improvement along a fixed and shared standard set by the goodness fixing kind, we have to misinterpret people’s judgements (e.g. they really thought – or at least should have thought - the G.38 is bad) or incriminate their epistemic standing (they were confused about the standards that apply to the G.38). Neither is a plausible option, especially given Thomson’s key epistemological claim (see Sect. 3.1). People praised the G.38, and rightly so. It really was the best civil aircraft at the time.

24 For reference, compare with our formulation of type (2) properties above: (2) Being good as an instance of a kind that may or may not be goodness-fixing in light of the standards for the goodness of a goodness-fixing kind.

25 Thanks to an anonymous referee for suggesting this point to us.
